# Supplementary material for: Solitary-specific drinking to cope motives explain unique variance in solitary drinking behavior but not alcohol problems compared to general drinking to cope motives
Source: PLoS One. 2023 Apr 13;18(4):e0282506. doi: 10.1371/journal.pone.0282506 (PMC10101636; doi:10.1371/journal.pone.0282506)
Supplement: S1 Table — (PDF) [file pone.0282506.s001.pdf]

S1 Table. Ranges and skewness/kurtosis values for all study variables.

Variable	Range	Skewness	Kurtosis
1. Drinking quantity	1-10	0.99	0.82
2. Drinking frequency	2-10	0.10	-0.77
3. Percentage of solitary drinking	1-100	0.75	-0.51
6. Solitary coping motives	1-5	0.60	-0.82
7. Solitary enhancement motives	1-5	0.71	-0.38
8. General coping motives	1-5	0.46	-0.76
9. General enhancement motives	1-5	0.14	-0.94
11. YAACQ-B	0-24	0.88	-0.04

Note. YAACQ-B = Young Adult Alcohol Consequences-Brief.
